# Impact of a Web-Based Lifestyle Medicine Intervention: A Qualitative Study Among Rural Participants

**DOI:** 10.3390/nursrep15070227

**Published:** 2025-06-25

**Authors:** Laurie Abbott, Jessica De Leon, Lucinda J. Graven

**Affiliations:** 1 College of Nursing, Florida State University, Tallahassee, FL 32306, USA; lgraven@fsu.edu; 2 College of Medicine, Florida State University, Tallahassee, FL 32306, USA; jessica.deleon@med.fsu.edu

**Keywords:** chronic illness, lifestyle, rural health, nursing

## Abstract

**Background**: Chronic diseases negatively impact health outcomes and are attributed to elevated morbidity and mortality throughout the world. Rural populations are disproportionately affected by chronic diseases and have limited access to health programs and resources. The purpose of this qualitative study was to explore the cognitive impact that a web-based lifestyle medicine intervention had on participants recruited from rural community settings in the southeastern United States. This qualitative study was the second phase of a mixed methods design, which used a randomized controlled trial to evaluate intervention effects. **Methods**: The descriptive design of this qualitative study included semi-structured interviews to collect information about the impact of a lifestyle medicine intervention. The interviews were transcribed verbatim and analyzed using the grounded theory method of thematic analysis in an iterative process for discovering patterns, central themes, and concepts. **Results**: The four themes that emerged were (a) gaining awareness and overcoming barriers, (b) encouraging others as a “ripple effect”, (c) realizing actualities, and (d) seeing progress. These themes highlighted the cognitive impact of an online lifestyle medicine program and provided insights about how rural dwellers perceived and processed educational health information. **Conclusions**: This study shows that the intervention had a positive influence on participants that continued after participation. The findings of this study provide recommendations that can facilitate intervention development and implementation among rural populations.

## 1. Introduction

Chronic diseases contribute toward the deaths of 43 million people around the world [1], resulting in a significant public health crisis. Chronic diseases are defined as noncommunicable conditions that require ongoing medical treatment and limit activities of daily life [2]. In the United States (U.S.), chronic diseases such as cardiovascular disease, stroke, and cancer are the primary causes of elevated rates of mortality and morbidity as well as high annual healthcare costs exceeding $4.5 trillion in national healthcare dollars [2,3]. In the U.S., approximately six out of ten adults live with one chronic disease, and four out of ten are managing two or more, with rates anticipated to rise as people live longer lives, regardless of progressive advances in medical care [2,3,4,5]. Age and family history are nonmodifiable chronic disease risk factors, but health behaviors, such as poor nutrition, lack of exercise, and tobacco and alcohol use, are modifiable disease risk factors that contribute to more than half of all preventable disease deaths [2,4].

Rural populations in the U.S. are disproportionately burdened by chronic diseases, including cardiovascular disease, stroke, hypertension, diabetes, and cancer, and are more likely to have worse health and die prematurely [6,7,8]. Research indicates that health outcome differences between rural and urban groups have increased over the past thirty years and are expected to continue widening in the future [9,10]. Higher comparative chronic disease rates among rural groups are associated with modifiable risk factors including unhealthy diet, obesity, physical inactivity, alcohol and tobacco intake, and deficient sleep [6,7,8,11]. Rural chronic disease disparities are exacerbated by social determinants of health, including limited access to healthcare services and lower socioeconomic and education levels [2,4,8].

Public health programs that address multiple chronic disease risk factors are scarce in rural populations [8]. Interventions guided by a lifestyle medicine approach based on the six pillars identified by the American College of Lifestyle Medicine (ACLM) have the potential to mitigate chronic disease disparities through health education and motivational methods based on consuming a mainly plant-based diet, regular exercise, adequate sleep, management of stress, avoidance of risky substance use, and positive social connections [12,13,14,15]. Lifestyle medicine interventions are cost-effective and have been shown to improve health behaviors, reverse disease progression, and reduce chronic disease development and exacerbation [2,5,16,17,18,19].

Information about the impact of lifestyle medicine interventions among rural groups is scarce, as recent lifestyle medicine studies have primarily discussed risk factor modification outcomes within urban samples [20,21,22,23]. To help bridge this literature gap, the purpose of this study was to explore the perceptions of rural participants about participating in a web-based lifestyle medicine intervention. The findings can enhance understanding of the program’s impact on rural dwellers and facilitate the development and tailoring of future chronic disease risk reduction programs.

## 2. Materials and Methods

The qualitative descriptive design of this study used a grounded theory process for exploring the cognitive impact experienced by rural participants of an online lifestyle medicine intervention. The study was approved by the institutional review board (IRB) at Florida State University, and informed consent was obtained before any study activities were conducted. The qualitative study reported here was the second phase of a sequential mixed methods design that had a preliminary quantitative phase that tested lifestyle medicine intervention using a randomized controlled trial among participants (n = 80) randomized to either intervention (n = 40) or control (n = 40) groups. The program had six group-based sessions about chronic diseases and disease risk factors based on the six pillars delineated by the ACLM [12].

### 2.1. Sample

The participants were recruited from rural areas in the southeastern United States. Those eligible to participate in the study were adults older than 18, proficient in the English language, and self-identified as having at least one chronic disease risk factor or diagnosis, including hypertension, being overweight or obese, smoking history, chronic disease family history, etc. Eligibility also involved being able to access the online platform used in the study, having either a smartphone with cellular data or a computer with internet service, and residing in a rural county that was defined by the Rural-Urban Continuum Codes (RUCC). RUCC codes describe counties as either rural or urban by criteria such as the size of the population, degree of urbanization, and distance to a metropolitan location [24]. Purposive sampling methods were used in recruiting participants from the intervention group after they completed sociodemographic and baseline surveys, intervention sessions, and three post-intervention surveys.

### 2.2. Data Collection

Appointments were made with participants who were willing to provide perspectives about the impact following program participation. The study began in September of 2022, and data collection ended in May of 2024. Online individual semi-structured interviews were conducted in the summer of 2023 by the three authors, who were similarly experienced in interviewing techniques for qualitative study data collection. Participants were asked to find a quiet place in their homes to avoid potential distractions and influences from other family members. The individual interviews began by asking participants about their overall view of the program, followed by more focused questions guided by a semi-structured interview guide. The interview guide included questions to elicit perceptions about ways participation in the program might have impacted their understanding of chronic diseases and their associated lifestyle risk factors. For example, questions included “Did the sessions help you to better understand chronic disease risk factors such as physical inactivity, being overweight, poor nutrition, etc.? Why or why not?” and “Have you applied any of the strategies to reduce disease risk to your everyday life?” The interviews were closed captioned (CC) and recorded within the online platform, and the baseline transcripts were the CC-captured text transcripts. Trained project personnel reviewed all audio/video interviews to finalize and clean the verbatim transcripts. Potentially identifiable information from the recordings was excluded from the written transcripts, including names of individuals or specific geographic locations. After the interview, each individual participant received a $50 gift card to a superstore.

### 2.3. Analytic Strategy

The thematic analysis was guided by the grounded theory approach for analyzing qualitative data. Grounded theory was the chosen method to understand the unique perspectives of rural program participants and elicit themes that can be practically applied in future public health program planning [25]. Data analysis was assisted by entering de-identified interview data into analytical software for qualitative analysis (NVivo 20 and Taguette (https://www.taguette.org/)) to facilitate data coding and categorization. An iterative process of open, axial, and selective coding enabled the discovery of patterns, concepts, and central themes using methods to ensure credibility and trustworthiness [26]. A codebook was developed to include parent codes, sub-codes, descriptions, and inclusion details (Table 1). Inter-rater reliability was enhanced by periodic meetings between the first and second authors to reach consensus regarding coding processes and codes, as well as discrepancies. Data saturation in qualitative research ensures that data collection continues until no new themes or patterns are noted, and for grounded theory research designs, recommended sample sizes are typically between 20–30 participants [27]. Data analysis was finalized once code saturation and, because of variability in experiences, a full understanding of participant perspectives was reached for meaning saturation at 26 participants (Table 1), and no other meanings, nuances, or insights were noted [28].

## 3. Results

The sample (Table 2) included a subset (n = 26) of intervention group participants who were mostly females (n = 22, 84.6%). The average age was 59.38 (+ 14.99) years, and most self-identified their race as African American (n = 9, 34.6%) or White (n = 15, 57.7%). Most participants were also employed part-time (n = 8, 30.8%) or full-time (n = 11, 42.3%). Many were married (n = 13, 50%) and had graduated from college (n = 12, 46.2%). From the list provided in the sociodemographic survey, a majority (n = 20, 76.9%) were previously diagnosed with 1–2 chronic diseases, and many people (n = 14, 53.9%) indicated they had five or more personal chronic disease risk factors.

**Figure 1:**
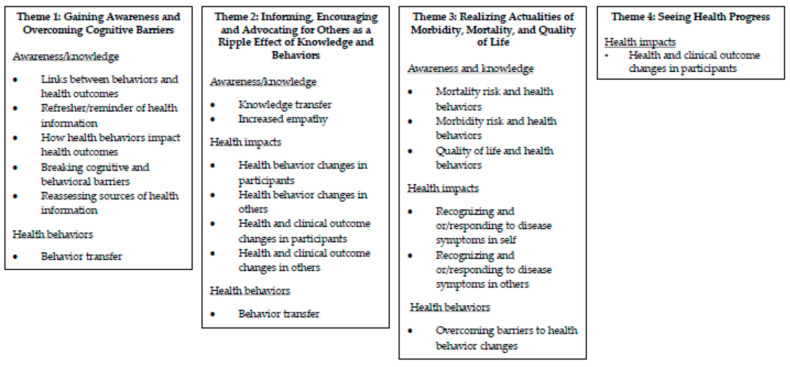
Themes and Associated Codes.

The results of the analysis included four themes using associated codes: “Gaining Awareness and Overcoming Cognitive Barriers”, “Informing, Encouraging, and Advocating for Others are a Ripple Effect of Knowledge and Behaviors”, “Realizing Actualities of Morbidity, Mortality, and Quality of Life”, and “Seeing Health Progress” (Figure 1).

The “Gaining Awareness and Overcoming Cognitive Barriers” theme contained experiences of gaining a greater understanding of chronic diseases and modifiable risk factors because of increased knowledge following program participation. Also encompassed were perceptions of linking health information together and making decisions that the present was the right time to make health behavior modifications. The “Informing, Encouraging, and Advocating for Others as a Ripple Effect of Knowledge and Behaviors” theme included insights regarding the impact of program participation on other people. The “Realizing Actualities of Morbidity, Mortality, and Quality of Life” theme included life realities associated with chronic diseases. The “Seeing Health Progresses” theme involved making progress and sensing health results. The four themes were categorized by the identification numbers of participants using codes, subcodes, and examples of participant quotations (Table 3).

### 3.1. Gaining Awareness and Overcoming Cognitive Barriers

Participants reported increased awareness and understanding of the impact of the program’s messaging on health behavior in various ways. The program enhanced understanding of the connections between health behaviors and health conditions by illuminating linkages between them. Program participants also reported that the program provided another level of contextual understanding by teaching them about the scientific and physiological context of health and illness, helping them comprehend the rationale behind the health advice they receive.

*So, a lot of (the health advice) we already do, but now we know why.*

(1)

Some entered the program with prior knowledge of chronic disease and modifiable risk factors or had already been working on healthier lifestyles. They reported the program was still beneficial as a refresher of their health knowledge and reinforcement of evidence-based guidance on positive health behaviors. Participants also conveyed that the program served to refresh old knowledge gained in the past, as well as replace past perceptions or behaviors that are now regarded as unhealthy. An informant said the scientific and evidence-based information provided in the program made her reconsider and reexamine her health information sources.

*I have another book I was going to mention in the class that says to “eat a lot of fat”, and just decided I’m going to go with (the information from) this class. I’m going to take the information from this class and apply it, and I’m not going to read it (the other book).*

(46)

Participants also reported that the program provided them with a greater realization of the severity or negative impacts and consequences of their health behaviors, leading to a breakdown of cognitive and behavioral barriers participants had been experiencing.

*…before, I didn’t realize how bad your eating habit was. Getting in the program and doing the session…it just opened my eyes, and kind of broke down a mental barrier.*

(35)

Participants who were already consciously working on positive health behaviors stated that the program made them more consistent and committed to performing positive health behaviors. One participant relayed a mental change regarding physical activity.

*(The program) made (me) more faithful, faithful in doing something, as far as exercise… you need to get up and do something, and it just got my attention. I think that was very beneficial for me.*

(13)

### 3.2. Informing, Encouraging, and Advocating as a “Ripple Effect” of Knowledge and Behaviors

A valuable program benefit was that the enhanced knowledge and improved behaviors of participants had a beneficial “ripple effect” on family and social networks via diffusion of information or the inclusion of others in the health behavior changes. Participants stated that modeling healthier lifestyles encouraged others to work toward making modifications as well. They also mentioned advocating for others and supporting those with chronic health conditions. For example, an informant discussed how learning more about chronic diseases stimulated “sympathetic” and “empathetic” feelings toward people living with the burden of chronic health conditions. In this case, the increased awareness, empathy, and sympathy led to advocacy for smoke-free environments in support of those with asthma. Other participants discussed involving others in behavioral changes. One participant relayed how her workplace enacted various health promotion programs because of the shared information and its influence on motivating coworkers to improve their health.

*We did a thing where we can see who lost the most weight and who does the most steps. We did it for two months, and each pay period we had to put in $20. At the end of that two months, which was like a week ago, I actually was the one out of eight people who lost the most weight. I lost a total of six pounds, and then I was the one that averaged out to 8- or 9000 steps. It ended at the end of May, and we’re going to start it back up in July, because everybody in the building has been struggling with losing weight. So, when I was breaking everything down to my director, she was like, “You know what, we’re going to apply what you learned, and all of us are going to work together to lose weight.” So, we got a little thing going.*

(3)

### 3.3. Realizing Actualities of Morbidity, Mortality, and Quality of Life

Although involvement in the program facilitated the acquisition and sharing of knowledge regarding disease etiology, the literal “life and death” impact on participants was even more meaningful. The combination of enhanced clarity about the extremely serious consequences of negative health behaviors and increased knowledge led participants to better recognize acute events such as strokes and heart attacks. Informants stated that the program information they learned could be “life-changing for anybody”, and some expressed that it was actually life-changing by empowering participants with evidence-based knowledge and behavioral strategies for themselves and others to live healthier and longer lives. The participants also described that the program made them realize the serious and potentially deadly results of unhealthy behaviors and ineffective chronic disease management. The program was described as “a wakeup call” to focus on health, reduce morbidity and mortality, and sustain quality of life.

*…now, (I am) 55, almost 56 in a month. I got to get my butt moving, or I’m going to be in a pine box before you know it.*

(17)

One of the most consequential benefits of the program was providing participants with the information and tools to recognize symptoms of serious health conditions. Many participants had family members with chronic and serious health conditions and felt the program content could help them recognize and address these health issues. Participants also expressed that the knowledge gained from participation in the program motivated necessary health behavior changes. Several informants relayed stories about applying new knowledge gained while in the program and afterward that facilitated recognition of alarming symptoms in themselves and others, including family members, friends, and coworkers. As these stories show, their subsequent actions and interventions, which may not have occurred prior to the educational intervention, had potentially life-saving results:

*…I really believe this class probably saved my husband’s life for us not to waste time, because he didn’t have much more time to get to the hospital. These sessions have, I mean, it saved his life, bottom line, no questions asked.*

(56)

### 3.4. Seeing Health Progresses

The time frame between the conclusion of the program and the follow-up interviews allowed participants to have a sense of progress from their efforts in making health behavior changes. They reported that they were making progress with health behavior changes and noticing measurable results. For example, participants stated they had purposefully lost weight since starting the intervention after learning about the effect of being overweight and obesity on increased chronic disease risk. Some mentioned that they had markedly lower joint pain and improved blood pressure readings, which influenced the lowering of antihypertensive medication dosage. The participants stated that the progress they made improved overall health, was recognized by primary healthcare providers, and enhanced quality of life.

*I feel like my doctors were pleased too, to see the progress, and it feels like we both feel like I’m getting better on track. Mentally, I feel better too.*

(36)

## 4. Discussion

The results of this study provided insight into the cognitive impact on health behavior change experienced by rural participants of an online lifestyle medicine intervention developed to reduce chronic disease risk and exacerbation. Consistent with previous research, the findings suggest that preventive health programs, including the six ACLM pillars, that address modifiable and contributory chronic disease risk factors positively influence health [12,29]. However, investment in prevention is minimal despite its potential to reduce chronic disease morbidity, mortality, and rising treatment costs [4]. This lack of public health services and resources is especially problematic for rural and remote populations around the world. Research about the effect and impact of online public health interventions is needed to potentially expand services in underserved areas [30].

A key theme that emerged from the study results was that participants gained an increased awareness of the relationships between lifestyle choices and health outcomes. Heightened awareness of one’s subjective experiences and actions can serve as a catalyst by inducing mental changes in how unhealthy behaviors are personally evaluated, leading to sustainable changes [31,32]. A deeper understanding of the impact of behavioral risk factors on the development and progression of chronic diseases empowered participants to make informed health decisions. For some participants, the program reinforced health behaviors already made, but for most, increased health literacy from program participation increased understanding and spurred intentions to engage in recommended lifestyle behaviors. This is especially important in rural populations where health literacy levels are typically low [33]. Health literacy entails health knowledge, motivation, and capabilities to comprehend and subsequently apply health information to health decisions and daily life activities [34]. Thus, interventions that improve health literacy in rural populations are vital in reducing chronic disease risk and fostering healthy habits throughout the life course.

Participants experienced mental changes, overcame existing cognitive barriers, and were more committed and intrinsically motivated in making healthy lifestyle choices. Learning about the long-term effects of unhealthy lifestyle behaviors, such as linkages between high dietary fat intake and heart disease, enhanced perceptions of personal risk that led to increased motivation to modify health habits. Gaining knowledge about chronic diseases and related causative risk factors promoted greater awareness of adverse disease outcomes and successively fostered the self-efficacy necessary for changing health behaviors. Previous research supports the importance of self-efficacy in promoting individual determination toward making positive lifestyle changes [35,36]. In fact, self-efficacy is crucial in maintaining optimal health, with evidence supporting its importance in managing chronic diseases, including cardiovascular disease [37] and diabetes [38], both of which are prevalent diagnoses among rural populations [9].

The second theme revealed that the benefits of the program extended beyond the individual participant to family members, friends, and colleagues. Social support is a known beneficial facilitator for health program participants that promotes improved outcomes and sustainability of healthier behaviors [39]. Notably, behavioral interventions can leverage social networks to aid intervention uptake [40]. However, in this study, the lifestyle changes that participants were making had a “ripple effect” that also benefited family, even children in the family group, as well as social and workplace networks through the modeling and sharing of program information. This finding is like that of previous studies that showed the reach of behavioral interventions extending beyond study participants to others in social networks [41,42], garnering a wider influence, which is particularly relevant in rural populations where participation in lifestyle management interventions may be low.

The third theme highlighted the meaningful effect that participants experienced greater realization about the actual threats posed by chronic diseases and unhealthy risk factors, and their association with increased disability and premature death. Enhanced awareness of different diseases, such as heart disease and stroke, and risk factors simultaneously served as both a warning and a motivating factor toward taking preventive action. In contrast to pharmaceutical treatments that target specific disease processes, lifestyle interventions can have a cumulative effect of simultaneously impacting multiple disease risk factors, improving and sustaining overall health, and reducing adverse effects over time [43]. Many participants expressed the desire to maintain health and live longer lives with loved ones and to assist with caring for children and grandchildren. These findings are important because they can facilitate understanding about the drivers of family-focused chronic disease risk reduction and outcomes [44].

The fourth theme accentuated that study participants sensed progress in their health, especially reductions in weight and blood pressure. Improving overall health and outcomes aligns with national goals for avoiding premature death and attaining longer life spans free of preventable chronic diseases [45,46]. Learning about chronic diseases, risk factors, and prevention strategies during the intervention motivated participants to actually make lifestyle changes that showed measurable progress. Although modifying habits can be challenging, setting specific goals and suggesting realistic strategies that support lifestyle goal achievement enhance self-efficacy among program participants in making strides toward clinically significant health behavior changes [47,48].

### 4.1. Strengths and Limitations

The study had strengths and limitations. A strength was the data collection method, using individual interviews to capture perspectives without influence from other program participants. The qualitative semi-structured interview design provided depth and insight regarding the cognitive impact experienced by participants. A limitation was that the sample included mostly women. Unfortunately, as in this study, men are less likely to engage in and are often underrepresented in health research [49], and future studies could strategize about methods to increase participation by men in preventive health programs and explore gender differences in perceptions about the cognitive impact of participating in health interventions and other associated outcomes. The qualitative design of the research, while providing depth and detail, limits generalizability. However, applying the findings to other rural settings in both high-income and low-to-middle-income countries can be feasible depending upon how researchers determine the “fittingness”, or comparability, with similar settings and groups [50].

### 4.2. Implications for Practice and Future Research

The findings of this study have practice implications for public health professionals on the front lines in clinic and community health settings to help facilitate the development of tailored interventions for improving health behaviors, advancing health, and reducing rural disparities. Future research can also explore the impact of programs on family and other social networks to detect intervention extension effects, especially among underserved groups with children. Promoting healthier lifestyle choices can facilitate health behavior changes that persist throughout the life course.

## 5. Conclusions

This study contributed new insights to support the importance of public health programs in mitigating chronic diseases and contributory risk factors. The four themes highlighted the cognitive impact of an online chronic disease reduction program and provided insights about the ways rural dwellers perceived and processed educational health information. With the high prevalence of chronic diseases and associated risk factors found among rural populations, interventions are needed to mitigate consistently poorer outcomes reported throughout the world. These findings suggest that lifestyle medicine programs are promising strategies for influencing healthier behaviors among people living in rural areas.

## Figures and Tables

**Table 1:** Program Impact Codebook.

Parent Code	Subcode	Description	Inclusion Details
Awareness and knowledge	New	Program content provided new awareness or knowledge to participant	Completely new information or filling in knowledge gaps
	Refresher/reminder	Program content refresher or reminder of participant’s current awareness or knowledge	Refresher of known health information
	Reassessing sources of health information	Participant reports reassessing sources of health information	Focusing on evidence-based health information
	Reassessing health beliefs	Participant reports reassessing their health beliefs	Reassessing past beliefs
	Knowledge transfer	Participant reports sharing program information with others	“Ripple” effect of information outflow to familial networks, social networks, and others
	Behavioral risk factors	Participant reports health behavior awareness/knowledge	Sedentary lifestyle, substance use, diet and nutrition, sleep
	How health behaviors impact health outcomes	Participant reports greater awareness and understanding of how and why health behaviors impact health outcomes	Understanding science, anatomy, physiology,
	Morbidity risk and health behaviors	Participant expresses increased understanding/greater awareness of the significant impacts of health behaviors on morbidity	Heart attack, stroke, diabetes in self or others (e.g., family members)
	Mortality risk and health behaviors	Participant expresses increased understanding/greater awareness of the significant impacts of health behaviors on mortality	Extending life for family, retirement,
	Increased empathy	Increased empathy for those with chronic diseases	Expressions of empathy
Health Behaviors	Overcoming barriers to health behavior changes	Breaking personal cognitive and behavioral barriers to change	Initiating change, motivating change, taking a first step, getting out of “comfort zone”
	Health behavior changes in participants	Participant reports changes in their health behaviors	Increased physical activity, substance use reduction, sleep, dietary changes, increased health screening/monitoring, increased knowledge of the health system
	Behavior transfer	Participant reports that their behavioral changes have transferred to or impacted others	Increased physical activity, substance use reduction, sleep, dietary changes, increased health screening/monitoring Others include participants’ kin networks, social networks, or others
Health Impacts	Health and clinical outcome changes in participants	Participant report changes in health and clinical outcomes	e.g., weight loss, blood pressure reduction
	Health and clinical outcome changes in others	Participant reports changes in the health and clinical outcomes of others	e.g., weight loss, blood pressure reduction Others to include participants’ kin networks, social networks, or others
	Recognizing/responding to symptoms in self	Participants report recognizing and/or responding to disease symptoms in themselves	e.g., stroke symptoms, heart attack symptoms
	Recognizing/responding to symptoms in others	Participants report recognizing and/or responding to disease symptoms in others	e.g., stroke symptoms, heart attack symptoms Others to participants’ kin networks, include social networks or others

**Table 2:** Sociodemographic Characteristics.

Participants (n = 26)	n	%	M	SD
Age (years)			50.38	14.99
Gender				
Male	4	15.4		
Female	22	84.6		
Employment				
Full-time	11	42.3		
Part-time	8	30.8		
Not working and looking for a job	1	3.8		
Not working and not looking for a job	1	3.8		
Retired	3	11.5		
Full-time Homemaker	2	7.7		
Marital Status				
Married	13	50.0		
Not Married/Have a Significant Other	4	15.4		
Widowed	2	7.7		
Divorced/Separated	7	26.9		
Education				
GED/High School Diploma	4	15.4		
Attended some college	10	38.5		
College Graduate	12	46.2		
Race				
White	15	57.7		
African American	9	34.6		
Other	2	7.6		
Number of People in Household				
1–2	15	57.7		
3–4	8	30.8		
5 or more	3	11.5		
Income				
Under $30,000	10	38.5		
$30,000–$49,999	5	19.2		
$50,000–$74,999	3	11.5		
$75,000–$100,000	3	11.5		
Over $100,000	5	19.2		
Risk Factors for Chronic Diseases (n)				
1–2	6	23.1		
3–4	6	23.1		
5 or more	14	53.9		
Personal History of Chronic Diseases (n)				
1–2	20	76.9		
3–4	5	19.2		
5–6	1	3.8		

**Table 3:** Themes, Code, Subcodes, and Significant Quotations.

Theme 1: Gaining Awareness and Overcoming Cognitive Barriers			
Participant	Code	Subcode	Example Quotes
19	Awareness/knowledge	Links between behaviors and health outcomes	I think we forget about how everything crosses over. How if you have high blood pressure, if you have diabetes, lack of movement, not eating well... how so many things play into our health… I think we just take it for granted, we don’t think about it.
73	Awareness/knowledge	Links between behaviors and health outcomes	(The program) made me think about my general health and well-being, and the risk factors associated with what we do and what we eat.
36	Awareness/knowledge	Links between behaviors and health outcomes	…it just clarified how it all correlates and connects together.
13	Awareness/knowledge	Refresher/ reminder	It was so long ago that we learned it, or we’ve never used it. …it’s a gentle reminder of you got it in your head—it’s time for you to start doing it. So I think it was very worthwhile.
19	Awareness/knowledge	Refresher/ reminder	I have been on a new, healthy lifestyle change for probably about three years now. So, it was just good to have some things reinforced.
19	Awareness/knowledge	Refresher/ reminder	I’m a nurse, so I’ve taken nutrition, so I’ve known…but it helps kind of like as a refresher…The last time I worked in nutrition was years and years ago, so even though it’s something that I knew, it was a good refresher to kind of remind me.
39	Awareness/knowledge	Refresher/ reminder	Some of the numbers that are considered pre-hypertension and hypertension, that was a refresher. I had heard it before, but again, if you don’t use certain things, you may not think of it as clearly.
19	Awareness/knowledge	Refresher/ reminder	(When) we were growing up in the nineties, it was all about ‘eat as little as possible, or ‘do this 3-day diet, lose ten pounds’. So, it’s just good to have reinforced as to what’s healthy and what to do, because sometimes, even though you know better, because it’s what you grew up with and what you tend to know, it’s what you tend to go back to. …it’s just good to have something to kind of refresh like, hey, this is the way can we do this right.
46	Awareness/knowledge	How health behaviors impact health outcomes	The saturated fat, and realizing what happens in your body, exactly what’s going on. …the fat and the arteries and heart disease—just learning the science of the heart disease and learning what you can do and feeling like there are things that you can do to help. …just to know what is really happening in your body, that helped a lot.
59	Awareness/knowledge	How health behaviors impact health outcomes	It’s something that I haven’t paid attention to very well in my life and something that I want to (pay attention to) more. I didn’t really realize that it was so much a health factor. … I didn’t realize how much it’s going to also impact my health. Just doing better, and I guess that’s why you feel better. I should have put those together, but really, being part of this program helped me have a better understanding and awareness of that... and being in a direction I’m glad I’m going.
46	Awareness/knowledge	How health behaviors impact health outcomes	(The interventionist) talked about blood pressure and that was really eye-opening about how, why that happens.
13	Awareness/knowledge	Breaking cognitive and behavioral barriers	(Program) put it to the forefront. It’s making you think about it. You think—you just don’t do things rote anymore, you do things outside of your comfort zone.
8	Awareness/knowledge	Breaking cognitive and behavioral barriers	I think I finally made that step—this is it. You really need to buckle down and get healthy.
46	Health behaviors	Behavior transfer	I think it was extremely helpful for us, and helpful, because I’m always the one like, let’s do this…He’s doing it now for his own, not because of me, but because of himself.
**Theme 2: Informing, Encouraging, and Advocating for Others as a Ripple Effect of Knowledge and Behaviors**			
**Participant**	**Code**	**Subcode**	**Example Quotes**
3	Awareness/knowledge	Knowledge transfer	After each section, I was able to call my Mom, and then call my grandparents, and explain to them what I had just learned about, and it’s actually helping.
73	Awareness/knowledge	Knowledge transfer	I learned something that I was able to share with someone, with other people, with my family in particular.
39	Awareness/knowledge	Knowledge transfer	I just talked to my aunt in Georgia this morning via telephone and I was telling her that we went through this study…and I told her about some of the things regarding the oils, the cooking oil.
1	Awareness/knowledge	Knowledge transfer	It just makes a world of difference. I was telling somebody the other day about what I learned, and so she’s going to start implementing it, because I told her, I feel so much better. I’m able to talk about it to others. (Others ask me) “but why are you going up the stairs?” “Well, let me tell you.” So, it is a door opener for me.
69	Health behaviors	Behavior transfer	I already read the labels, but if I just read the labels for myself and not for my family, and they grow up unhealthy, I’m not doing anything, anybody any good. So, thank you for that.
46	Health behaviors	Behavior transfer	I think it was extremely helpful for us, and helpful, because I’m always the one like, let’s do this…He’s doing it now for his own, not because of me, but because of himself.
73	Health behaviors Health impacts	Behavior transfer Health and clinical outcome changes in others	My wife’s a diabetic, and some of the diet changes and daily habits she’s changed, and she feels better, and I definitely feel better because of that.
3	Health impacts	Health behavior changes in participants Health behavior changes in others Health and clinical outcome changes in others	We all changed our eating habits. (The program) actually helped me so that I can help my 10-year-old. He’s 10, and he was 150 pounds in January. And now with me changing up everything, he’s now down to 130.
13	Awareness/knowledge	Increased empathy	It helped me to understand what they are going through. It helped me to understand and be more, not only cognizant, but maybe more sympathetic, empathetic. …when I hear people who suffer with asthma, and they talk about their medication, that little pump that they take, and going around cigarette smoke. I empathize with them, and whether they are there or not, I do bring up smoke free environments. So, it just made me more caring, exhibiting more care of what people are going through.
**Theme 3: Realizing Actualities of Morbidity, Mortality, and Quality of Life**			
**Participant**	**Code**	**Subcode**	**Example Quotes**
48	Awareness/knowledge	Mortality risk and health behaviors	(The program) was probably hitting us at the right time, and I really appreciate the opportunity. It was good for me. I think it might have even extended my life. I have children, and I have grandchildren. So, I want to stay around.
17	Awareness/knowledge	Mortality risk and health behaviors	…now, (I am) 55, almost 56 in a month. I got to get my butt moving, or I’m going to be in a pine box before you know it.
69	Awareness/knowledge	Mortality risk and health behaviors	I had my children late in life, so ... I’m 64 and the grandchildren are just now coming. I have two grandchildren and one step grandchild, and I just want to be there.
25	Awareness/knowledge	Morbidity risk and health behaviors	(The program made) you more aware of the (health) consequences of not doing it, you know what I’m saying? And seeing what can happen.
35	Awareness/knowledge	Mortality risk and health behaviors Morbidity risk and health behaviors	Being knowledgeable in that kind of stuff just helps you to provide a better life for yourself, to help you live longer and healthier.
28	Awareness/knowledge	Mortality risk and health behaviors Morbidity risk and health behaviors	(The program) shows you the importance of not overdrinking, smoking, not exercising, eating the wrong things. It all plays (into) if you want a long life. Who wants to have a stroke? So, all those things are important.
49	Awareness/knowledge	Morbidity risk and health behaviors	My father actually had a heart attack like a week after the sessions ended. …I can use this knowledge now because we have to eat heart healthy. Once it hits home, then you realize, oh, yeah, we actually need to start doing this. …these sessions I’ve just done, and then the heart attack... I was like, we know how to eat healthy now, and we know why we need to. That way we don’t have to go through this again. So, I would have to say (the program sessions) are definitely useful.
36	Awareness/knowledge	Morbidity risk and health behaviors	…this is really important. This is something that I really got to take seriously because number one, I don’t like that I don’t have energy. I don’t like the way I feel sometimes with my joints and everything. I think about my kids who are just now 18 and 20. One day they are going to get older. They are going to have kids of their own. How am I going to have the energy to take care of them? …I really want to start putting more into place so that way I can live to be an old age and enjoy life. So, the sessions, they kind of were a little bit of a wake-up call.
49	Awareness/knowledge	Morbidity risk and health behaviors	…you have the information, but unless it really hits home, or you realize, if you don’t eat (better), (heart attack) is going to happen to you or to your loved one.
72	Awareness/knowledge	Morbidity risk and health behaviors	Sit down and listen about some things that’s been going on with your health or somebody in your family. It was helpful, all of it, to me, because my husband is a diabetic, my mom had a stroke. It’s just different things that went on with my family.
48	Awareness/knowledge	Quality of life and health behaviors	Let’s face it. We all want to live longer, and I want to make it the best last quarter of my life, or whatever it may be. I want to do it as good as I can.
35	Health impacts	Recognizing and/or responding to disease symptoms in others	I keep a closer eye out. That way, if I see any signs that anybody, or myself, is having something like a heart attack or a stroke, or anything like that, I can know what to do and how to do it.
36	Health impacts	Recognizing and/or responding to disease symptoms in others	My aunt actually had one while I was doing this session. She had a stroke. I kept thinking she might be having symptoms of a pre-one. And luckily her home health nurse showed up the day that she was having a stroke. It was less than fifteen minutes.... The session on strokes happened right within like a few days of that happening to her. So, it helped me understand it a little bit better, as well, of what was happening with her. My aunt was having the slurred speech…the left lacks moving, like her motor functions weren’t as good... and we recently had somebody that was experiencing that at work, but I didn’t know about it at the time, but later... And what they told me, I was like, well, that’s a stroke. The hospital sent them away though at first, was saying it was something else.
56	Health impacts	Recognizing and/or responding to disease symptoms in others	A couple of weeks ago, my husband actually showed signs of a heart attack, and it was through this program that I was like, “No, we can’t wait. We have to go now.” He actually had to have a triple bypass. It was because of the class that kind of made me know the signs of when not to wait, when it is okay to wait. But his skin turned gray. He was kind of slurred in his talking. They said he actually had a mild heart attack on the way there.
69	Health impacts	Recognizing and/or responding to disease symptoms in self	My ankles were swelling, but only one of them. My heart and my chest were tightening. I ended up with an EKG because I was concerned it was a blood clot. Otherwise, normally, I would not have even gone. I would have put my feet up. I would have just tried to bring my pressure down. I was truly concerned for a blood clot. I knew it was heart related. So now I’m moving to the next phase of the treatment where I’m actually going to a cardiologist.
3	Health behaviors	Overcoming barriers to health behavior changes	…after the sessions, when I received all the information, I seen the importance of it, that I really needed to do it. That kind of gave me that boost that I really needed. Okay, I need to make these life changes.
**Theme 4: Seeing Health Progress**			
**Participant**	**Code**	**Subcode**	**Example Quotes**
36	Health impacts	Health and clinical outcome changes in participants	I’m happy to say since our sessions I’ve lost 4 pounds.
73	Health impacts	Health and clinical outcome changes in participants	I’ve lost 18 pounds.
3	Health impacts	Health and clinical outcome changes in participants	Since the sessions, I have lost 10 pounds.
1	Health impacts	Health and clinical outcome changes in participants	Oh, my blood pressure! I went back to the doctor. At one point my blood pressure was 152 over 84, and it’s down to 104 over 78.
3	Health impacts	Health and clinical outcome changes in participants	My doctor was able to lower my blood pressure medicine because of all the helpful information that I received.

## Data Availability

Most qualitative data relevant for this study and findings are available in the manuscript’s Results section and Table 2.

## Public Involvement Statement

The public was involved in this research by responding to recruitment and outreach methods, providing informed consent, and voluntarily participating in the study.

## Guidelines and Standards Statement:

The reporting of this research was guided by the SRQR (Standards for Reporting Qualitative Research: A Synthesis of Recommendations) qualitative study method.
